# Inhibition of Src phosphorylation reduces damage to the blood-brain barrier following transient focal cerebral ischemia in rats

**DOI:** 10.3892/ijmm.2014.1946

**Published:** 2014-09-24

**Authors:** YONGSHENG BAI, GUANGHUI XU, MENGXUE XU, QI LI, XINYUE QIN

**Affiliations:** Department of Neurology, The First Affiliated Hospital of Chongqing Medical University, Chongqing 400016, P.R. China

**Keywords:** vascular endothelial growth factor A, blood-brain barrier, claudin-5, middle cerebral artery occlusion, tight junction

## Abstract

The disruption of the blood-brain barrier (BBB) caused by cerebral ischemia determines the extent of injury and patient prognosis. Inhibitors of Src can markedly minimize the infarct size and preserve neurological function. The Src protein tyrosine kinase (PTK) inhibitor, PP2, protects the rat brain against ischemic injury, possibly through the reduction of vascular endothelial growth factor A (VEGFA) expression and the upregulation of claudin-5 expression, which preserves the integrity of the BBB. In this study, the expression levels of phosphorylated (p)-Src, VEGFA and claudin-5 were determined to investigate the changes occurring in the levels of these proteins and to determine the benefits of PP2 treatment following cerebral ischemia/reperfusion (I/R). Our study included a sham-operated group, an I/R group, a vehicle-treated group (V) and a PP2-treated group (PP2). We found that the rats in the PP2 group exhibited greater preservation of neurological function and reduced VEGFA and p-Src protein expression compared with the rats in the I/R and V groups. Moreover, the mRNA and protein levels of claudin-5 were markedly higher in the PP2 group than in the I/R group or the V group after 3 days of reperfusion. Immunofluorescence staining revealed that the co-localized immunostaining of fibrinogen and claudin-5 was reduced in the PP2 group, which suggests that the exudation of fibrinogen in this group was less than that in the I/R and V groups. Furthermore, the reduced co-localization of immunostaining of glial fibrillary acidic protein (GFAP) and claudin-5 indicated that the rats in the PP2 group had only a slight disruption of the BBB. These findings suggested that PP2 treatment attenuated the disruption of the BBB following ischemia and minimized the neurological deficit; these effects were associated with a decreased VEGFA expression and an increased claudin-5 expression. Members of the Src PTK family may be critical targets for the protection of the BBB following cerebral ischemia.

## Introduction

Cerebral edema is a leading cause of mortality after an ischemic stroke. The disruption of the blood-brain barrier (BBB) caused by cerebral ischemia can initiate the development of cerebral edema and the progression of brain injuries. The disruption of BBB integrity is one of the most crucial initiating factors in cerebral ischemia/reperfusion (I/R) injury (1). Water content progressively increases within 2 days of an occlusion and then gradually decreases for up to 14 days following an ischemic stroke (2). The vascular leakage associated with an ischemic insult may lead to a narrowing of the vascular lumen and to an increase in the viscosity of the blood, which results in the progression of brain edema and in the impairment of microvascular perfusion, which in turn contribute to the subsequent neuronal injury (3). It has been known for several years that vascular endothelial growth factor (VEGF) is a secreted mitogen that is associated with angiogenesis and is also a potent vascular permeability factor (4,5). In rats, transient and permanent middle cerebral artery (MCA) occlusions initiate the expression of VEGF in the ischemic brain (6–8). Astrocyte-derived VEGFA has been shown to be involved in BBB leakage (9). The early post-ischemia administration of recombinant human VEGF (rhVEGF) to ischemic rats has been shown to significantly increase BBB leakage, hemorrhagic transformation and the size of ischemic lesions (10). In the ischemic brain, the inhibition of VEGF during the acute stage of a stroke may reduce the permeability of the BBB and the risk of hemorrhagic transformation following focal cerebral ischemia.

Src family kinases serve as intermediate steps in several cell signaling pathways and propagate signals directed toward the cytoskeleton, membrane and nuclear targets. Following a cerebral ischemic injury, continuous cytokine and growth factor stimulation can overactivate Src (11,12). Additionally, a previous study demonstrated that a Src inhibitor was effective when injected as late as 6 h after an ischemic injury (13). In that study, the inhibition of Src did not affect the resting cerebral blood flow or the perfusion of the ischemic hemisphere after the stroke (13). Accumulating evidence suggests that activated Src kinase regulates VEGFA production through the signal transducer and activator of transcription 3 (STAT3) pathway. Constitutively activated STAT3 has been reported to upregulate VEGFA expression and thereby induce tumor angiogenesis (14); phosphorylated Src thus aggravates BBB leakage following ischemia through the upregulation of VEGFA expression.

Claudin-5 in endothelial cells is a major structural component of the BBB formed by cells within the neurovascular unit (NVU). Claudin-5 may play a key role in the formation of the paracellular pores or channels that mediate the selective ion permeability of the BBB (15). It has previously been reported that dynamic changes occur at the level of tight junction protein expression in the BBB in response to traumatic brain injury. VEGFA specifically downregulates claudin-5 protein and mRNA expression in cultures of brain microvascular endothelial cells (16). In the mouse cerebral cortex, the microinjection of VEGFA has been shown to disrupt claudin-5 and induce the loss of barrier function. The authors of that study suggested that the downregulation of claudin-5 by VEGFA is the mechanism for the BBB breakdown (16). PP2 is a Src protein tyrosine kinase (PTK) inhibitor that decreases the infarct volume and improves neurological scores following MCA occlusion (26). These data suggest that the VEGFA-mediated decrease in claudin-5 may be involved in the disruption of the BBB following ischemia. The protective effects of PP2 during ischemic injury are thus due to the increase in claudin-5 expression and the subsequent protection of the BBB.

However, the mechanisms through which PP2 reduces infarct size and preserves neural function remain unclear. In the present study, we evaluated the protective effects of PP2 on the BBB during an ischemic insult. We aimed to perform an extensive analysis of the changes occurring in the protein levels of VEGFA, phosphorylated (p)-Src and claudin-5 and in the mRNA levels of claudin-5 and to evaluate the effects of PP2 on claudin-5 protein expression and the leakage of fibrinogen through the BBB following I/R injury in rats.

## Materials and methods

### Reagents and antibodies

The Src PTK inhibitor, 4-amino-5-(4-chlorophenyl)-7-(t-butyl)pyrazolo[3,4-d]pyrimidine (PP2), was obtained from Merck Calbiochem Co. (Darmstadt, Germany). The primary antibodies used were rabbit anti-Src and anti-p-Src (pY418) (Epitomics, an Abcam Co. Burlingame, CA, USA), mouse anti-VEGF (Santa Cruz Biotechnology, Inc., Santa Cruz, CA, USA), claudin-5 monoclonal antibody (CLDN5; Invitrogen, Life Technologies, Carlsbad, CA, USA), rabbit anti-glial fibrillary acidic protein (GFAP; Boster, Wuhan, China) and rabbit polyclonal antibody to β-actin (Beijing Biotech Co., Ltd.). The following secondary antibodies were used: Alexa Fluor 594- conjugated AffiniPure goat anti-rabbit IgG, Alexa Fluor 488- conjugated AffiniPure goat anti-mouse IgG [Beijing Zhongshan Golden Bridge Biotechnology Co. Ltd. (ZsBio), Beijing, China], goat anti-rabbit or goat anti-mouse secondary antibody (Histostain-Plus kits; ZsBio). A QuantiFast SYBR-Green PCR kit (Qiagen, Hilden, Germany) was used for the quantitative (real-time) polymerase chain reaction (PCR) assays.

### Animals

All the experimental procedures were approved by the Administrative Panel on Laboratory Animal Care of Chongqing Medical University (no. SCXK2007-000341). A total of 155 Sprague-Dawley rats (weighing 250–280 g) were used in this study. The rats were randomly divided into the following 4 groups: the sham-operated group (Sham), the I/R group, the vehicle (DMSO)-treated group (V) and the PP2-treated group (PP2). Transient middle cerebral artery occlusion (MCAO) was induced via intraluminal nylon filament intrusion as previously described by Longa *et al* (17). Briefly, the rats were anesthetized with an intraperitoneal injection of 3.5% chloral hydrate (350 mg/kg). A midline incision was made in the neck, and the right external carotid artery (ECA) was sequentially exposed and dissected. The distal portion of the ECA was ligated with sutures, and the branches between the ECA and ICA were also cauterized. After an incision was made in the ECA, a monofilament nylon suture was inserted from the ECA into the right internal carotid artery to occlude the origin of the right MCA. The sham-operated rats underwent identical surgeries with the exception that the suture was not inserted. The rectal temperature was maintained at 37.0±0.5°C with a heating pad and a heating lamp. Laser-Doppler flowmetry (Perimed, Stockholm, Sweden) was used to confirm the induction of ischemia and reperfusion in the rats. The Src family tyrosine kinase inhibitor, PP2, was dissolved in saline containing 1% dimethyl sulfoxide (DMSO). The PP2-treated rats were administered PP2 (1.0 mg/kg) (18), and the vehicle-treated rats were administered the same volume of the vehicle (DMSO) in the peritoneal space after 30 min of MCAO. After 120 min of occlusion, the suture was removed to allow reperfusion, the ECA was ligated and the wound was sutured.

### Neurological evaluation

The neurological function of each animal was assessed using a set of modified neurological severity scores (mNSSs) at 1, 3, and 7 days post-reperfusion. The mNSS is a composite measurement of motor, sensory, reflex and balance statuses (19). The neurological deficit was graded on a scale of 0 (normal) to 18 (maximal deficit). One point was awarded for the inability to perform the test or for the lack of a tested reflex. Therefore, higher scores indicated a more severe injury.

### Quantitative reverse transcription PCR (RT-qPCR)

The peri-infarct tissues that were supplied by the MCA were excised from the brain tissue on ice, snap-frozen in liquid nitrogen and stored at −80°C. Total RNA was isolated using TRIzol reagent (Takara, Dalian, China) according to the instructions of the manufacturer. With a PrimeScript RT Reagent kit (Takara), 1 μg of RNA was reverse transcribed, and genomic DNA was eliminated by the addition of DNase. The primers for the PCR assays were supplied by Sangon Biotech (Shanghai, China) and were as follows: claudin-5, 5′-GGCGATTACGACAAGAAGAACT-3′ (sense) and 5′-CCCGAACCCAACCTAACTT-3′ (antisense); β-actin, 5′-CCCATCTATGAGGGTTACGC-3′ (sense) and 5′-TTTAATGTCACGCACGATTTC-3′ (antisense). RNA was quantified using the QuantiFast SYBR-Green PCR kit (Qiagen). The PCR assays were performed in an Eppendorf Mastercycler PCR system according to the following protocol: 5 min hold at 95°C, followed by 10 sec at 95°C and 30 sec at 60°C (40 cycles). The transcript levels were standardized with β-actin and calculated using the ΔΔCT method.

### Western blot analyses of proteins and brain microvasculature fractionation

The ischemic brain tissue and time-matched tissue samples from the sham-operated rats were dissected. The protein samples were prepared using a commercially available kit (Beyotime). The proteins were separated by 10% SDS-PAGE and transferred onto polyvinylidene fluoride membranes. The membranes were blocked with 5% BSA for 1 h and were then incubated overnight at 4°C with primary antibodies. The membranes were washed prior to incubation with the secondary antibodies for 75 min at 37°C. After extensive washing, specific immunoreactivities were visualized via a chemiluminescence ECL plus system and quantified by scanning densitometry with a Bio-Image Analysis System (Bio-Rad, Hercules, CA, USA). Isolation of the brain microvasculature fraction was performed using a modified procedure that was similar to that of a previous study (20). The brain tissues from the ischemic hemispheres were homogenized in 5 ml Dulbecco’s modified Eagle’s medium (DMEM) and centrifuged at 3,000 rpm for 5 min. The homogenates were resuspended and then treated with 0.005% (wt/vol) dispase at 37°C for 2 h. The homogenates were centrifuged at 3,000 rpm for 5 min, and the pellets were re-suspended in a dextran solution (Mw 70,000; 10% wt/vol; Sigma) and centrifuged at 3,000 rpm at 4°C for 10 min. The pellets were then re-suspended in phosphate-buffered saline (PBS) and centrifuged at 1,000 rpm for 5 min. The final pellet was re-suspended in RIPA lysis buffer (50 mM Tris, pH 7.4, 150 mM NaCl, 1% Triton X-100, 1% sodium deoxycholate, 0.1% SDS, 2 mM sodium pyrophosphate, 25 mM β-glycerophosphate, 1 mM EDTA, 1 mM Na_3_VO_4_, and 0.5 μg/ml leupeptin) and centrifuged at 12,000 rpm for 20 min at 4°C; the supernatant was removed for claudin-5 analysis.

### Immunohistochemistry (IHC) and immunofluorescence

At various time points following reperfusion, the rats were deeply anesthetized with chloral hydrate and perfused through the left ventricle with ice-cold PBS and 4% paraformaldehyde. The brains were removed and post-fixed in 4% paraformaldehyde. The brain tissues were embedded in tissue-freezing medium and sectioned into 10 μm-thick slices. The remaining brain tissues were paraffin-embedded and cut into 7 μm coronal serial sections. For IHC staining, endogenous peroxidase was blocked by incubating the slides with 3% hydrogen peroxide for 20 min. The sections were rinsed 3 times for 5 min each with PBS. Non-specific binding sites were blocked by incubation with 10% control goat serum for 30 min. The brain slices were incubated with primary antibodies overnight at 4°C. The slides were rinsed 3 times for 5 min each with PBS and then incubated with secondary antibodies at room temperature for 30 min. The sections were rinsed 3 times for 5 min each with PBS and then incubated with an avidin-HRP complex solution for 30 min. The staining was visualized by developing the sections in a solution containing 5% 3,3′-diaminobenzidine (DAB) and 3% hydrogen peroxide in PBS. The sections were counterstained, dehydrated and mounted with coverslips. Sections that were stained without primary antibodies were used as negative controls. Five non-overlapping images in the peri-infarct area were acquired and analyzed using Image Pro Plus 6.0 software to measure the optical density of the positively stained areas.

Double immunofluorescence labeling was performed for claudin-5/fibrinogen and claudin-5/GFAP. The brain cryosections (10 μm) were pre-incubated in cold acetone and then rinsed in PBS 3 times for 5 min each. Following heat-induced antigen retrieval in a microwave in a citrate buffer (pH 6.0), the sections were blocked with 10% goat serum and were then incubated with a combination of mouse anti-claudin-5 antibodies and rat anti-GFAP antibodies or rabbit anti-fibrinogen antibodies and mouse anti-claudin-5 antibodies at 4°C. The sections were then rinsed and incubated with a mixture of Alexa Fluor 594 goat anti-rabbit IgG and Alexa Fluor 488 goat anti-mouse IgG for 2 h at room temperature. Images were acquired using a confocal microscope (Leica TCS Sp2; Leica Microsystems, Wetzlar, Germany). Lesions that were dual-labeled for claudin-5 and fibrinogen or GFAP were used to quantify the number of vessels in the entire lesion area that exhibited BBB breakdown. The data are presented either as percentages of the area of double-positive claudin-5 and fibrinogen staining compared to the total area of claudin-5 positivity or as percentages of cells that were double-positive for claudin-5 and GFAP compared to the total number of GFAP-positive cells.

### Statistical analyses

The values are presented as the means ± standard deviation (SD), and statistical analyses were performed using a one-tailed unpaired t-test for 2 groups or an ANOVA for comparisons of multiple groups. Statistical analyses were performed on the normalized real-time PCR and western blot analysis data to determine significant differences. An ANOVA and Tukey’s post-hoc test were conducted to determine the differences in the neurological scores. A value of p<0.05 was considered to indicate a statistically significant difference.

## Results

### Improvement of the neural deficit scale scores in the PP2-treated group

**Figure 1:**
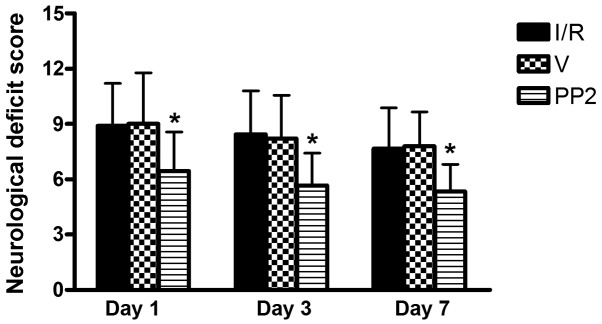
Effects of PP2 on neurological dysfunction following cerebral ischemia. Modified neurological severity scores (mNSSs) of the animals that were subjected to different ischemic insults. The PP2 group exhibited lower scores than did the ischemia/reperfusion (I/R) group or the vehicle (DMSO)-treated (V) group at 3 time points (^*^p<0.05 vs. the I/R group and vs. the V group; n=6 in each group.

To assess the immediate impact of PP2 treatment on functional recovery, we evaluated the average mNSSs for the Sham, I/R, V and PP2 groups at 1, 3 and 7 days following reperfusion. In the Sham group, no neurological deficit was found. No significant differences were observed between the I/R group and the V group at any of the examined time points. At 1 day post-reperfusion, the scores in the I/R, V and PP2 groups indicated moderate injuries; however, the PP2 group had a significantly lower mean score (6.4±2.01) than did the I/R and V groups (8.9±2.3 and 9.0±2.17, respectively, p<0.05). On days 3 and 7, the neurological scores of the PP2 group remained significantly lower than those of the I/R and V groups (Fig. 1).

### Expression of p-Src protein and the effects of PP2 on p-Src expression

**Figure 2:**
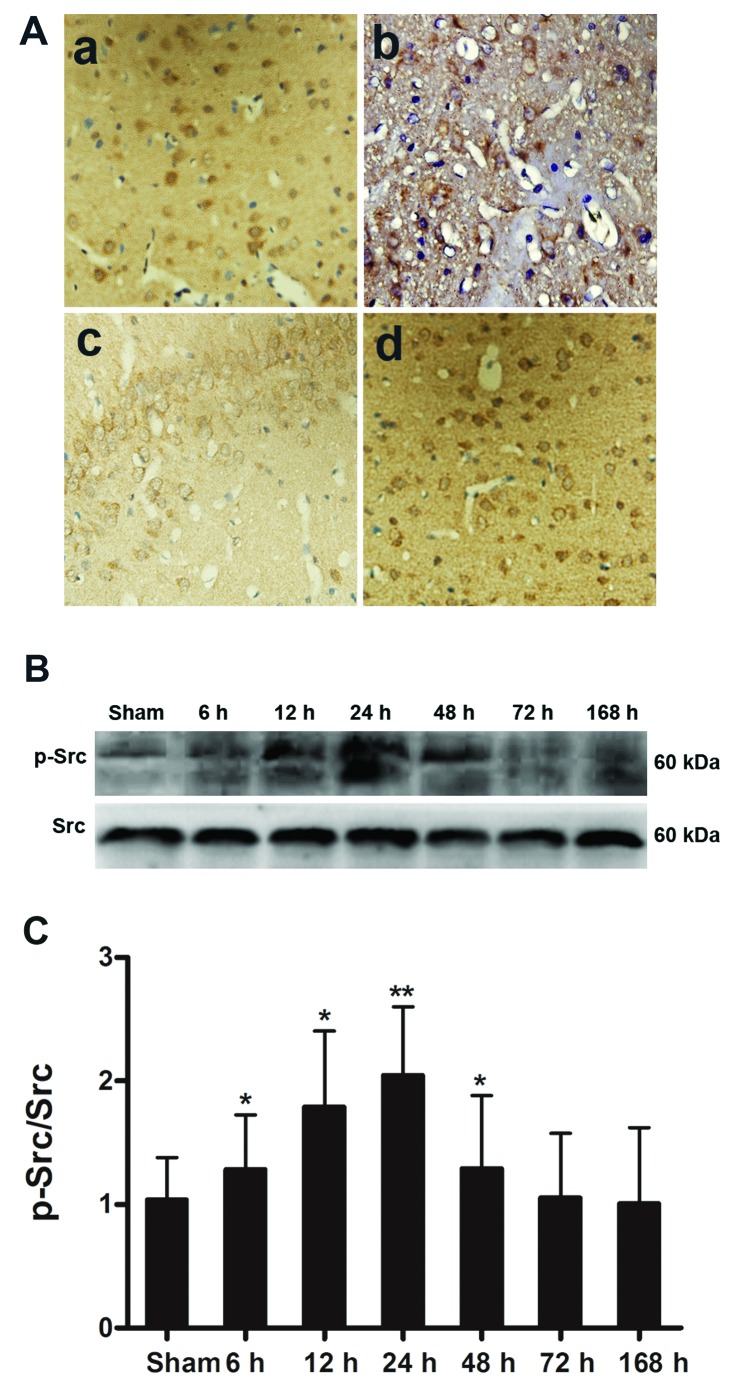
Distribution and expression of the Src protein analyzed by immunohistochemistry and analysis of the expression of p-Src protein by western blot analysis following ischemia. (A) (a-d) Src immunoreactivity was detected in the cortical neurons, astrocytes and perivascular tissues of rats in the sham-operated (Sham), ischemia/reperfusion (I/R), vehicle (DMSO)-treated (V) and PP2-treated (PP2) groups following reperfusion. (d) The Src-immunopositive cells in the PP2 group did not differ from those of the Sham or other groups. (a) Sham group; (b) I/R group; (c) V group; (d) PP2 group. Original magnification of the images, ×400. (B) Ipsilateral hemisphere tissue at various time points (Sham, 6, 12, 24, 48, 72 and 168 h) following ischemia and reperfusion. p-Src production was determined by western blot analysis. (C) The data are displayed as the values corresponding to the p-Src bands normalized to the total Src in the same blots. p-Src protein levels began to increase at 6 h, peaked at 24 h, and remained elevated until 48 h (^*^p<0.05 and ^**^p<0.01, significant differences compared to the Sham group, n=6).

**Figure 3:**
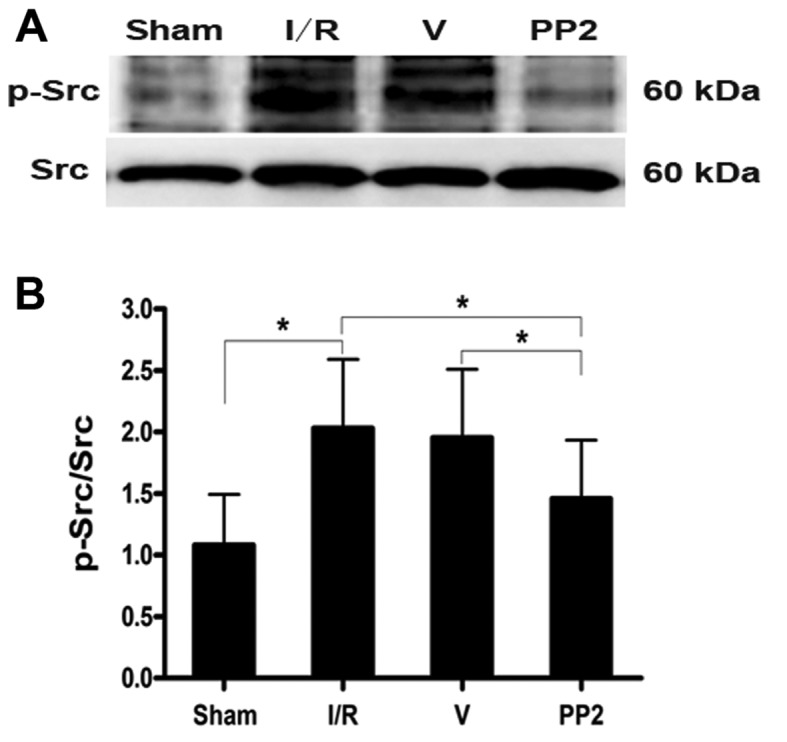
The increase in p-Src protein levels that was induced by ischemia/reperfusion injury was blocked by the Src inhibitor, PP2. (A) Representative immunoblots of p-Src from the 4 groups indicating that p-Src expression was reduced by PP2 in the PP2 group at 24 h following ischemc stroke. (B) Quantitative determinations of the immunoblots revealed that p-Src expression was strongly downregulated by PP2 in the PP2-treated group (^*^p<0.05, n=6; Sham, sham-operated group; I/R, ischemia/reperfusion group; V, vehicle (DMSO)-treated group; PP2, PP2-treated group).

Abundant Src immunoreactivity was detected (Fig. 2A) in the neurons of both the cortices and hippocampi of the animals in the Sham, I/R, V and PP2 groups (Fig. 2A-a–d, respectively) and can be seen as the yellowish-brown color in the micrographs. We also observed Src immunoreactivity in the astrocytes and cerebral perivascular tissue. The distribution of Src immunoreactivity was not altered following cerebral I/R injury (Fig. 2A-b–d). The Src-immunostained cells did not accumulate in the peri-infarct areas (Fig. 2A-b–d), and the Src-immunostained cells of the animals in the PP2 group did not differ from those of the animals in the Sham or the other groups (Fig. 2A-d). Western blot analyses were performed to further quantify the alterations in p-Src that were induced by I/R insults. p-Src was present at very low levels under normal conditions, and p-Src protein levels began to increase at 6 h, reached their peak value at 24 h, and remained at a high levels until 48 h after the ischemic insult. The tyrosine p-Src level in the rats in the I/R group was twice that of the rats in the Sham group at 24 h after the ischemic insults (Fig. 2B and C). Furthermore, the marked increase in p-Src levels that was induced by ischemia was significantly inhibited by PP2 at 24 h after the ischemic stroke. The PP2 group had a markedly lower level of the tyrosine p-Src than did the I/R and V groups (Fig. 3; p<0.05).

### Expression of VEGFA protein and the suppressive effects of PP2 on the expression of VEGFA after I/R injury

**Figure 4:**
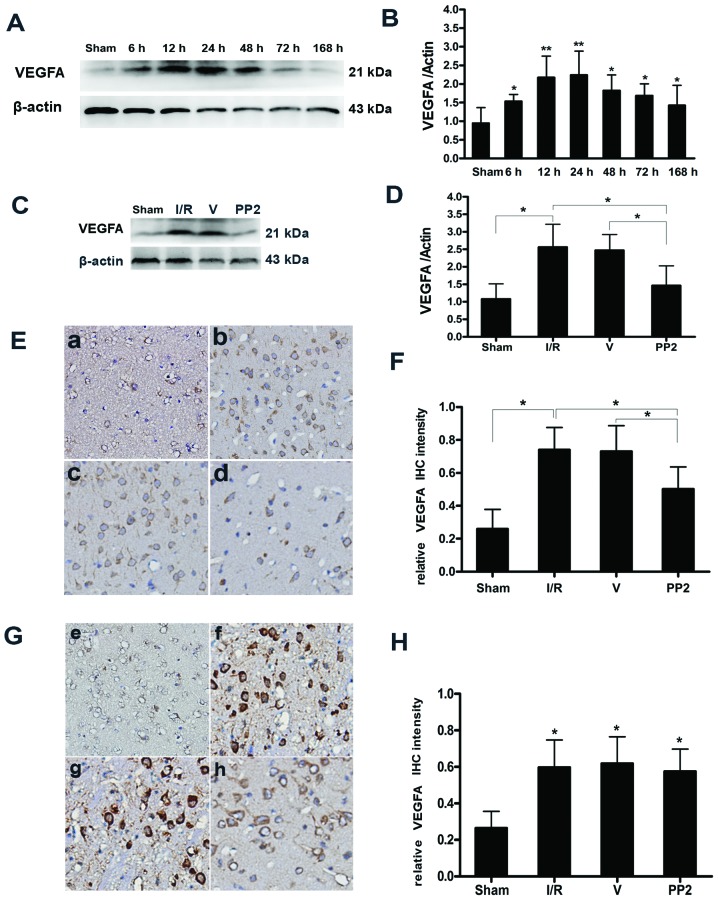
Expression of vascular endothelial growth factor A (VEGFA) protein and the suppressive effects of PP2 on the expression of VEGFA. (A) Representative western blot of VEGFA indicating that VEGFA expression was increased in the ipsilateral hemispheres at various time points (6, 12, 24, 48, 72 and 168 h) after ischemia/reperfusion (I/R) injury. (B) Values corresponding to the VEGFA bands were normalized to the sham-operated (Sham) group. VEGFA protein levels began to increase at 6 h, peaked at 24 h, and remained elevated until 7 days (^*^p<0.05 and ^**^p<0.01, significant differences compared with the Sham group, n=6). (C) Representative immunoblots of VEGFA from the Sham group, the I/R group, the vehicle (DMSO)-treated (V) group, and the PP2 group at 24 h following ischemic stroke indicating that the expression of VEGFA was strongly downregulated by PP2. (D) Quantitative determinations of the immunoblots revealed that VEGFA expression was markedly decreased by PP2 (^*^p<0.05, n=6). (E) Immunohistochemical staining for VEGFA in the penumbral areas at 24 h post-reperfusion. (F) Histogram of the optical intensities of the VEGFA-positive staining in the peri-infarct regions of the PP2 group were markedly decreased at 24 h (^*^p<0.05, n=6). (G) Immunohistochemical staining for VEGFA in the penumbral areas at 7 days post-reperfusion. (H) Histogram indicating that there were no differences in the optical intensities for VEGFA-positive staining in the peri-infarct regions of the ischemic hemispheres 7 days post-reperfusion across the 3 groups subjected to I/R (^*^p<0.05, n=6). (E and G) (a and e) Sham group; (b and f) I/R group; (c and g) V group; (d and h) PP2 group. The images shown in (b-d) were acquired 24 h after ischemia, and those in (f-h) were acquired 168 h after ischemia. The original magnification of the iamnges was ×400.

VEGFA protein expression was measured by western blot analysis and immunohistochemical staining as shown in Fig. 4A and E. We found that the VEGFA expression level was very low under normal conditions and that VEGFA immunoreactivity was observed in the astrocytes and peri-vascular tissue of the rats in the Sham group. VEGFA expression began to increase at 6 h, peaked at 24 h, and remained at a high level until 1 week following ischemic stimulation (Fig. 4B). The VEGFA protein level increased to 2-fold that of the Sham group at 12 and 24 h post-reperfusion, and the level of VEGFA at 24 h was slightly higher than that at 12 h (Fig. 4A and E). Furthermore, this increase in VEGFA protein expression was significantly inhibited by PP2 at 24 h after ischemia (Fig. 4C–F). The VEGFA expression level in the PP2 group was significantly lower than that in the I/R and V groups, and the number of VEGFA-positive cells in the PP2 group was much lower than that in the I/R and V groups. The intensities of VEGFA immunostaining did not differ between the I/R group, the V group and the PP2 group 7 days after ischemia. Increases in the VEGFA immunoreactivity in hypertrophic astrocytes were observed in the penumbra area at 24 h after ischemia, and these increases persisted for at least 1 week (Fig. 4E–H).

### Expression of claudin-5 and the effects of PP2 on the expression of claudin-5

**Figure 5:**
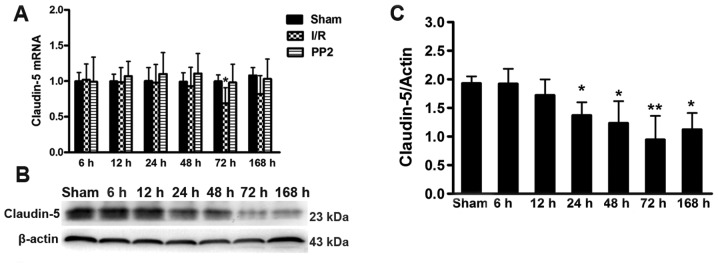
Evaluation of claudin-5 mRNA transcription and protein expression following ischemic injury and the effects of PP2 on claudin-5 transcription. (A) Claudin-5 transcripts were analyzed by RT-qPCR following ischemia/reperfusion (I/R) injury, and the effect of PP2 on the transcription of claudin-5 was assessed. The level of claudin-5 mRNA was significantly decreased at 72 h, and this decrease in claudin-5 transcription was markedly blocked by PP2 [^*^p<0.05 vs. the Sham-operated (Sham) group and the PP2 group at 72 h, ANOVA; n=5]; (B) Representative immunoblots for claudin-5 from the brain capillary protein fraction of rats in the Sham and I/R groups following reperfusion; ischemia induced a marked decrease in claudin-5 protein levels from 72 h to 1 week. (C) Values corresponding to claudin-5 bands revealed that the claudin-5 protein levels gradually reduced from 48 h to their lowest point at 72 h and remained at low levels until 1 week when compared with the Sham group (^*^p<0.05 vs. the Sham group at 6, 12 and 24 h; ^**^p<0.01 vs. the Sham group at 72 h, ANOVA; n=5).

**Figure 6:**
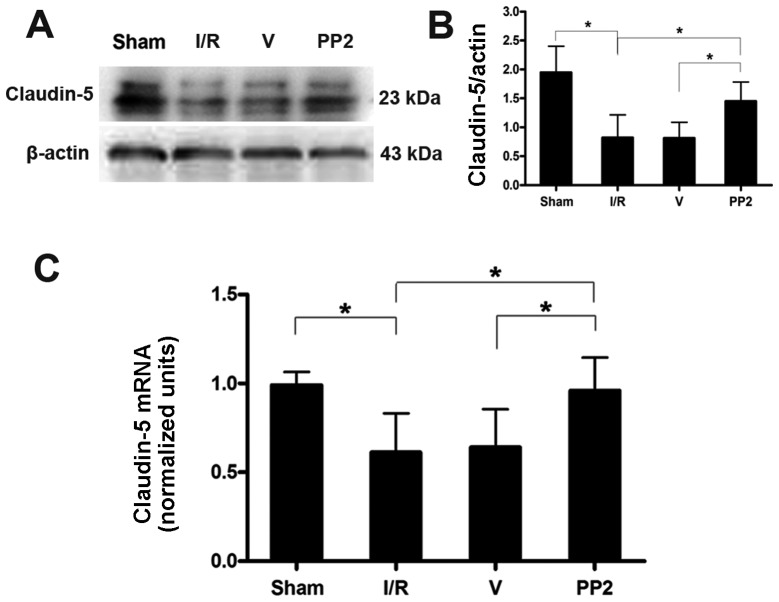
Protective effects of PP2 on claudin-5 expression. (A) Representative western blot from the Sham-operated (Sham), ischemia/reperfusion (I/R), vehicle (DMSO)-treated (V), and PP2 groups at 72 h after reperfusion. The decrease in claudin-5 protein expression was effectively prevented by PP2. (B) Quantitative determinations of claudin-5 protein expression revealed that PP2 markedly prevented the decrese at 72 h after I/R injury (^*^p<0.05, ANOVA; n=6). (C) Effects of PP2 on claudin-5 transcription were evaluated in the 4 groups at 72 h, and RT-qPCR revealed that the decrease in claudin-5 mRNA expression was significantly blocked by PP2 (^*^p<0.05, ANOVA; n=6).

Transcriptional analyses of the tight junction protein, claudin-5, were performed. An ischemic insult induced the inhibition of mRNA synthesis 72 h after the I/R injury. As expected, the Src inhibitor, PP2, blocked the decrease in the claudin-5 transcript level (Fig. 5C). Further experiments revealed that claudin-5 protein expression was consistent with the changes in the nucleic acid transcripts; however, the decrease in claudin-5 expression continued for 1 week following reperfusion (Fig. 5A and B). The results from RT-qPCR and western blot analysis were compared amongst all the groups at 72 h after the ischemic injuries. These comparisons demonstrated that PP2 prevented the decrease in claudin-5 protein and mRNA expression and protected the BBB (Fig. 6).

### Protective effects of PP2 on the BBB following ischemic insult

**Figure 7:**
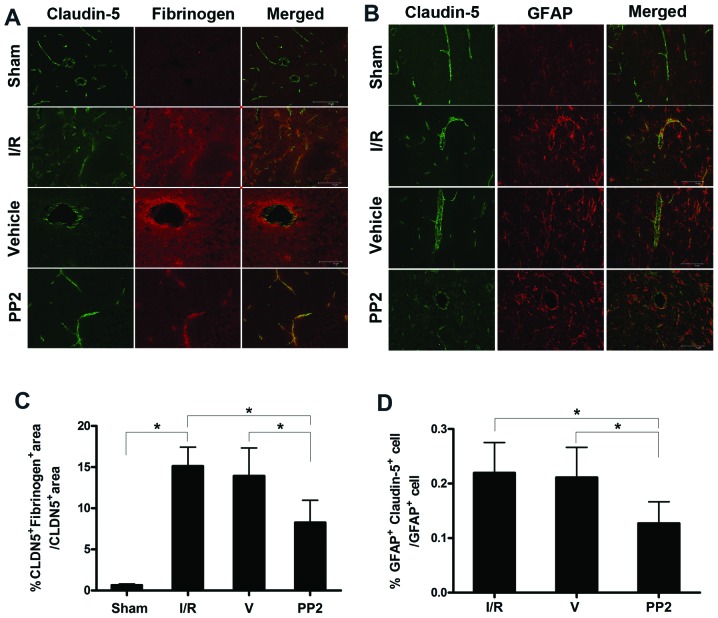
Protective effects of PP2 on endothelial tight-junctions. (A) Confocal micrographs revealing claudin-5 and fibrinogen immunoreactivity in the sham-operated (Sham), ischemia/reperfusion (I/R), vehicle (DMSO)-treated (V) and PP2 groups. The greater the fibrinogen extravasation in the I/R and V groups, the lower the claudin-5 levels, resulting in simultaneous severe blood-brain barrier (BBB) disruptions. The decrease in claudin-5 was blocked, and the extravasation of fibrinogen was markedly reduced, as observed in the confocal micrographs taken of the penumbral areas in the PP2 group 72 h after reperfusion. (B) Confocal micrographs from the Sham group indicating that the claudin-5 in the blood vessels was separated from the astrocytes; the confocal images from the penumbral areas of the rats in the I/R group reveal fragmentation and degeneration of the claudin-5 immunoreactivity. The merged immunostaining for claudin-5 and GFAP illustrates the close proximity of the astrocytic end-feet to the vessels in the I/R group. The proximity of the astrocytic endfeet to the vessels in the PP2 group was obviously decreased, and the integrity of the endothelial tight-junctions was significantly preserved. (C) The significant protective effect of PP2 on the BBB was confirmed via quantitative analysis (^*^p<0.05, ANOVA; n=6) (D) Quantitative analyses revealed that the double-staining for GFAP and claudin-5 in cells was markedly reduced by PP2 (^*^p<0.05, ANOVA; n=6) (scale bars, 75 μm).

As the disruption of the BBB that was induced by ischemia increased, claudin-5 expression decreased. Confocal imaging of the brain tissue from the rats in the Sham group revealed that the astrocytic endfeet around the blood vessels were completely separate from claudin-5, and no extravasation of fibrinogen was evident. The ischemic injuries induced the loss of claudin-5 immunoreactivity and increased the immunostaining for fibrinogen. When these changes were quantified, the analyses revealed a significant correlation between the disruption of the BBB in the rats following ischemic insult and the decrease in claudin-5 immunoreactivity; i.e., we found that the severity of the BBB disruption was proportional to the decrease in claudin-5 immunoreactivity, as well as to the increase in the extravasation of fibrinogen. Fibrinogen was primarily exuded at 72 h; PP2 protected the BBB from this disruption and significantly blocked the leakage of fibrinogen. The increased disruption of the BBB was accompanied by the co-localization of claudin-5 and GFAP in the ischemic hemisphere at 72 h. Quantitative analysis revealed a strong association between claudin-5 immunoreactivity and GFAP staining, and the more intense co-localization of claudin-5 and GFAP immunoreactivity correlated with more severe BBB disruptions. The increase in the co-localization of claudin-5 and GFAP was prevented by PP2; consequently, the integrity of the BBB was protected (Fig. 7).

## Discussion

*In vivo*, the BBB, with its neuronal and non-neuronal components, represents the NVU (21). The NVU is characterized by a functional interaction between the brain endothelial cells, astrocytes, pericytes, microglia and neurons. Ischemic injuries induce disruptions of the BBB that may cause numerous severe clinical complications, such as the formation of brain edema and neuroinflammation (22,23). Accumulating evidence indicates that tight junction proteins may play a pivotal role in the maintenance of the physical barrier function of the BBB (15,35). The Src family kinases mediate signaling activity in response to a variety of growth factors, including VEGF (24,25). Available evidence suggests that the Src kinase regulates VEGF-mediated vascular permeability in the brain following stroke and that the suppression of Src activity decreases vascular permeability and thereby minimizes brain injury. To assess the extent of the injuries in this study, we evaluated the average NSSs of the rats in the Sham, I/R, V and PP2 groups at 1, 3 and 7 days post-reperfusion; the Src inhibitor, PP2, markedly improved the neurological function of the rats in the PP2 group. No significant differences were observed between the I/R and V groups at the time points examined. The results of the present study are consistent with those of a previous study that found that Src inhibitors have neuroprotective effects (13). The protective effects of PP2 against ischemic injury may be mediated through its ability to protect the BBB and reduce the infarct area.

In the NVU, p-Src not only regulates cells, but also influences the surrounding environment. The results of our study revealed that Src was widely expressed in neurons, brain endothelial cells and astrocytes. p-Src is the activated form of Src and plays an important role in transcellular transduction. We observed that large quantities of Src were phosphorylated following ischemia, consistent with earlier observations. It has been reported that PP2 significantly reduces the infarct size compared with the controls following transient focal brain ischemia (26); however, the mechanisms underlying this effect have remained elusive. Takenaga *et al* (27) reported that the levels of p-Src reached their peak 6 h after ischemic injury; our study revealed that the time at which the levels of p-Src protein peaked was 24 h after ischemia. The ischemic penumbra is constantly changing and expanding from the central infarct area to the surrounding area. This discrepancy may be the result of differences in the sampling of the tissues examined. In our study, we examined samples of peri-infarct brain tissue that were distinct from the brain capillary fragments. Korematsu *et al* (28) reported that the occlusion of the rat MCA for 1 h led to a significant increase in protein tyrosine phosphorylation in the microglia in the insulted cerebral cortex 3 h post-reperfusion. Increased protein tyrosine phosphorylation has also been found in the rat retina following I/R injury, and the VEGF levels in the damaged tissue are also increased. Following an ischemic insult, astrocytes become hypertrophied and proliferative (29). It has previously been demonstrated that cellular (c)-Src is activated by the hypoxia-induced upregulation of VEGFA expression and that constitutive viral (v)-Src increases the VEGF mRNA levels (30). VEGFA was produced in response to an ischemic injury after p-Src was upregulated due to the hypoxemic stimulation (31). p-Src has been implicated in the production of astrocyte-derived VEGFA following ischemia (26,32). In our study, VEGFA was elevated at 6 h after reperfusion and peaked at 24 h, which was compatible with the changes in p-Src levels following the ischemic injury. Notably, recent studies have indicated that Src-induced VEGF upregulation requires the persistent activation of STAT3. For example, STAT3 has been shown to be necessary for VEGF upregulation through v-Src as the blockade of STAT3 signaling inhibits VEGF expression that is induced by Src tyrosine kinase activity (14). In the present study, the Src inhibitor, PP2, markedly reduced the production of p-Src and produced a similar inhibition in the increase in the VEGFA protein level. Quantitative analyses revealed that PP2 markedly decreased VEGFA expression, which verified that PP2 not only inhibited p-Src production, but also inhibited the expression of VEGFA. Immunohistochemical staining of VEGFA in the penumbral areas at 24 h post-reperfusion proved that VEGFA expression was markedly reduced; however, there were no differences in the optical intensities of the VEGFA-positive immunohistochemical stains in the peri-infarct regions of the ischemic hemispheres 1 week post-reperfusion among the 3 ischemic groups. Furthermore, our findings support the notion that PP2 inhibits the expression of VEGFA following ischemia. Our results revealed that the neurons and astrocytes had much stronger effects on the disruption of the BBB than did the endothelial cells, and these findings represent another discrepancy between this and previously reported data (27).

It is well established that claudin-5 is a pivotal component of the tight junctions between brain endothelial cells that effectively separate the brain from the cerebral blood flow. Claudin-5 was selected for our study due to previous observations that claudin-5 is present in large amounts in brain endothelial cells and is an endothelial cell-specific component of tight junction strands (33). Astrocyte-derived VEGFA has been implicated in the breakdown of the BBB during inflammation of the central nervous system. The VEGFA-induced downregulation of claudin-5 is a significant determinant of the increased paracellular permeability of brain microvascular endothelial cells (16). In the present study, we found that the claudin-5 mRNA levels in the rats in the I/R group were significantly lower than those of the rats in the Sham group at 72 h after ischemia; moreover, claudin-5 protein expression was downregulated from 48 h after the ischemic insult, and the decrease in claudin-5 protein expression was most evident at 72 h. The inhibitor PP2 blocked the downregulation of claudin-5 transcription at 72 h and similarly blocked the decrease in claudin-5 protein levels 72 h after ischemia. In transient ischemic experiments, Huang *et al* (34) observed that the opening of the BBB appears in the first half-hour of reperfusion; this is followed by a partial closing and then a delayed opening that occurs between 22 and 46 h post-reperfusion. In our study, a decrease in claudin-5 protein expression was detected at 48 h that was compatible with the findings of these studies. Although this bimodal increase in BBB permeability is widely believed to occur in the I/R model, the time points for the peak BBB permeability have not been completely defined. Jiao *et al* (35) found that 2 peaks in BBB permeability appeared after reperfusion, at 3 and 72 h post-reperfusion, following 2 h of focal ischemia. This result is consistent with our finding of a second BBB permeability peak at 72 h. However, Jiao *et al* (35) found a decrease in claudin-5 protein beginning at 6 h after reperfusion, which is different from our results. This difference may be attributable to the different tissue samples examined and the diverse trial methods employed. Liu *et al* (36) found that ischemia triggers 2 rapid and parallel processes that cause the early disruption of the BBB: matrix metalloproteinase-2 (MMP-2)-mediated occludin degradation and caveolin-1 (Cav-1)-mediated redistribution of claudin-5 from the cytoskeleton to the cytosol in the ischemic cerebral microvessels. During the early phase of BBB breakdown, Nag *et al* (37) observed a significant increase in caveolin-1 expression at the lesion site following injury at 12 h and on day 2, while claudin-5 expression was decreased only on day 2.

Fibrinogen leakage is a well-documented and validated marker of a breakdown in the BBB (38,39). In our study, confocal imaging of the peri-infarct regions of the ipsilateral hemispheres indicated that ischemia caused a loss of the claudin-5 immunoreactivity in the blood vessels and disrupted the appearance of the astrocytic foot processes around the endothelial cells. Following ischemia, claudin-5 immunostaining decreased, the BBB disruption increased and the extravasation of fibrinogen became more intensive and extensive; at 72 h, the extravasation of fibrinogen was massive. PP2 protected the BBB from disruption and significantly reduced the leakage of fibrinogen. Claudin-5 appeared in linear rows, which have previously been referred to as having a ‘zip-locked’ appearance (40). The astrocytic endfeet around the blood vessels were completely separated from the claudin-5 in the images from the Sham group. In the present study, we observed GFAP-positive immunoreactivity that co-localized with diffuse cytoplasmic claudin-5 immunostaining of the ischemic penumbra. The BBB was extensively disrupted, and the co-localization of claudin-5 and GFAP was obviously present in the ischemic hemisphere at 72 h. The observed fibrinogen leakage at 72 h supports the theory that the water content progressively increases within 2 days and that the second peak of opening the BBB appears within 3 days. PP2 protected the BBB from the disruption caused by VEGFA and decreased the co-localization of GFAP and claudin-5. PP2 markedly reduced the area of co-localization and significantly protected the BBB against ischemic insult.

The main causes of the delayed opening that occurred at 72 h were the VEGFA-mediated decrease in claudin-5 at the transcription and protein levels; however, other factors cannot be excluded. As previously demonstrated, since the inflammatory response that occurs secondarily to ischemia and hypoxia following reperfusion had fully developed, many additional factors were involved, including cytokines and infiltrating leukocytes (41). Therefore, the possibility that PP2 blocked MMP or eNO expression and activation cannot be completely excluded (29,42). Moreover, future studies are planned to elucidate the mechanisms of the effects of VEGFA on the expression and transcription of claudin-5. The BBB opening that occurs in claudin-5-deficient mice is selective for small molecules (<800 D) (43), whereas the BBB disruption that follows ischemia is more severe and less size-selective. The loss of claudin-5 is important for the initiation of barrier disruption *in vivo*, whereas the loss of occludin (OCLN) that has been reported (37,44) may exacerbate the phenotype. Our results suggest that the VEGFA-induced downregulation of claudin-5 constitutes an important mechanism of BBB disruption following an ischemic insult and that the inhibitor of Src exerts a protective effect against I/R injury. These findings may indicate a potential target for therapeutic intervention.
